# Clinical Observations on the Constitutional Origin of the Various Forms of Porrigo

**Published:** 1834-01-01

**Authors:** 


*Clinical Observations on the Constitutional Origin of the various Forms of Porrigo.* By George Macilwain, Surgeon to the Finsbury Dispensary.—London, 1833. 8vo. pp. 83.

Everyone who is acquainted with the minute anatomy of our profession must be aware that there is nothing by which young beginners are more harassed than by the unceasing importunities of their friends to write a book. It is in vain they plead that they know nothing, and have nothing to say. "Is there anything in Dr. Z.'s book on Indigestion?" reply their friendly tormentors, "and yet it brought him a host of patients; and have not the 'Catheterical Studies' of Mr. X.

though copied verbatim from Cooper's "Surgical Dictionary," procured him half the urethral practice of Wapping?" These counsels are the exciting causes of the birth of heaps of queer unread little books, whose appearance every publishing season is a subject of astonishment to the ignorant; and we confess that, had we not been informed by an advertisement that Mr. Macilwain is already the father of a book, we should have attributed the breaking-out of this work to some such irritating applications. As the matter stands, we cannot conceive the motive for publishing these "Clinical Observations;" for not only any physician or surgeon, but any intelligent attorney, surveyor, or engineer, might have informed our author that there is nothing in them: anyone accustomed to read and write would have told Mr. Macilwain that the frog emulating the ox was not in a more desperate condition than the author, who, with matter scarcely sufficient for a letter to a periodical, has determined to blow-out a book of eighty-three pages.

The first chapter, which contains the introduction, has a very mysterious title: "The history of cutaneous affections shews them, for the most part, to be tractable in nearly the same proportion as they are unconstitutionally treated;" and the chapter itself is a very tough morsel. In order to enable our readers to judge of this matter, we will first translate the chapter out of Macilwainish into our own plain English, and then give it in the original tongue.*

Mr. Macilwain, then, declares that the more violent a cutaneous disease is, the more certainly and quickly it yields to medical treatment: a doctor is frightened by a scarlet fever, and drives it away in a prodigious hurry, but takes a porrigo very coolly, and, contented with anointing his patient's pustules, forgets that he has a stomach. Now we have always thought that the brief duration of the exanthemata did not depend on professional "anxiety, which renders us absolutely regardless of circumstances of minor import," but was a law of nature. A grandmother-nurse, rich in the accumulated absurdities of half a century, stifling her patients with blankets, and inflaming them with cordials, might prevent recovery or protract convalescence, but could never (so at least we thought,) produce a chronic scarlatina. Or, to take the other extreme in the scale of intellect, a Sydenham was satisfied with watching the course of a disease which no medi-

* At the end of this pamphlet there is a notice of Mr. Macilwain's first book, extracted from the *Foreign* Quarterly Review, promising to review it in their next number: meaning, you see, gentle reader, that it was not written in English.

cine could cut short, and has left us the caution, which every one recollects, against the *nimia medici diligentia*. But, lest we should be accused of breaking a butterfly upon a wheel, we will quote, without farther preface, the peccant chapter itself.

"In considering the probable progress of practice in so large a class of diseases as those affecting the skin, we should certainly *a priori* be disposed to anticipate that those diseases would be most intractable which were attended by the greatest constitutional disturbance, or which threatened inflammation or structural alteration of vital organs, and that, in proportion as a disease became ushered in or accompanied by little general disorder, so would it become amenable to the influence of treatment.

"The state of the fact, however, with regard to cutaneous diseases, is widely different; the facility with which the various forms yield to our art, being, with few exceptions, in a tolerably accurate proportion to the serious, extensive or complicated general disturbance by which they are characterised. This result, apparently so contrary to all rational anticipation, appears to me to admit of the following explanation. In some diseases the formidable nature of the general disturbance not only compels us to direct our attention to it, but often with an anxiety which renders us absolutely regardless of circumstances of minor import; as happens in acute forms of the exanthemata. Other diseases are too manifestly accompanied by a bad state of health to allow of their connexion with it to be overlooked or mistaken; a third set are so obstinate, so pertinaciously annoying, and are attended with so little local alteration, that this disproportion alone suggests the possible influence of a cause not to be discovered in the seat of its local manifestation. These circumstances (and as it appears to me, very much in the way here represented,) have led physicians and surgeons already to treat the majority of cutaneous diseases by means directed to the improvement of the general health.

"There are however still cutaneous affections in which the disturbance is neither formidable, alarming, nor very plainly developed, and in which its very existence, because often unattended by any very obvious indication, is overlooked and merged in the contemplation of the more prominently troublesome and disgusting peculiarities of their local characters, and wherein the treatment has been confined to the removal of these, regardless of the constitutional disturbance on which they depend. This is strongly exemplified in the treatment usually pursued in the various forms of Porrigo, and the result is just what sounder views of disease must lead us to expect; viz. that with the exception of specific malignant diseases, they are the most obstinate of all cutaneous affections.

"This appears to me to be a brief but true account of the state of the practice with regard to Porrigo; and I can only say, that if it be in any sense an overdrawn picture, it has not been occasioned by supineness on my part in endeavouring to ascertain the real state of the case. I have availed myself of every opportunity which has presented itself of inquiring into the practice of others, and at the same time freely communicated the nature and results of my own. I cannot add more on this head without subjecting myself to misconstruction, or, what would be worse, saying that which might not be agreeable to others. I shall therefore only hope, that the reader will give the treatment a fair trial, and assure him of my confidence that the result will not disappoint his expectations." (P. 5.)

The rest of the book may be comprised in two lines: Feed porriginous patients on vegetable diet; keep their bowels open; use the Ung. Hydr. Nitr., "variously diluted."

We are afraid that Finsbury critics have very sour tempers; for Mr. Macilwain, as we have seen, was obliged to put an untimely end to chapter i., lest he should subject himself to misconstruction, and say "that which might not be agreeable to others." Again, at page 51, after telling us that too much medicine is as bad as too much food, he says, "The observation may possibly subject me to some unpleasant criticism; but I cannot but think that some rules of a very elementary character are too frequently disregarded in medical treatment." And at page 24 he observes, "As I should scarcely be excused for publishing distinct cases of porrigo, I will now state the general results of the treatment of this disease in the Finsbury Dispensary," &c. The objectors to the publication of "distinct cases of porrigo" must be very crabbed mortals indeed; but, to shew Mr. Macilwain that we are not so cross-grained, we will part from him with the following advice. We do not think porrigo a bad subject for a book, but he has not hit the method of writing upon it. As he cures the disease quicker than other people, and has cases by wholesale, let him in future keep a register of them; let him print, in a tabular form, the names of the patients, their residences, ages, state when first seen, treatment employed, and result; and he will make more converts than by putting forth a dozen vague and verbose pamphlets, and calling them "Clinical Observations."

